# Oral Supplementation with Betaine Powder Ameliorated High Blood Pressure in Spontaneously Hypertensive Rats

**DOI:** 10.3390/metabo14070390

**Published:** 2024-07-18

**Authors:** Samanthi Wathsala Pelpolage, Rie Sasaki, Kenichiro Shimada, Taizo Nagura, Hirokatsu Uchino, Kyu-Ho Han, Michihiro Fukushima

**Affiliations:** 1 Department of Life and Food Sciences, Obihiro University of Agriculture and Veterinary Medicine, Inada, Obihiro 080-8555, Hokkaido, Japan; samanthiwp@obihiro.ac.jp (S.W.P.); stm5.amnos@gmail.com (R.S.); kshimada@obihiro.ac.jp (K.S.);; 2 Research Center, Nippon Beet Sugar Mfg., Co., Ltd., Obihiro 080-0831, Hokkaido, Japan; nagu@nitten.co.jp (T.N.);

**Keywords:** betaine, blood pressure, nitric oxide, angiotensin I converting enzyme

## Abstract

Supplementation of betaine is associated with improved cardiac health, potentially due to its function in re-methylation of homocysteine, an independent risk factor for cardiovascular diseases. We investigated the effects of oral betaine supplementation on blood pressure homeostasis in spontaneously hypertensive (SHR) rats and Wistar Kyoto (WKY) rats in an 8 week-feeding trial with control (SHR-con and WKY-con) and 1% betaine supplemented (SHR-b and WKY-b) diets. Systolic, diastolic, and mean blood pressure in the SHR-b group were significantly lower at week 8 (*p* = 0.013, *p* = 0.011, *p* = 0.010, respectively). Furthermore, serum nitric oxide (NO) levels were significantly (*p* < 0.05) improved in the WKY-b and SHR-b groups, suggesting a healthy endothelial function. Additionally, the serum angiotensin I converting enzyme level in SHR-b rats was also significantly lowered, which may have been another reason for lower blood pressure. A significantly higher non-HDL level in the SHR-b group might reflect enhanced lipid secretion into the circulation in the form of very-low-density lipoprotein (VLDL). Betaine is known for its effect on the synthesis of phosphatidylcholine, a key component of VLDL. However, the long-term net outcomes of both blood pressure lowering and serum lipid increment should be further studied.

## 1. Introduction

Maintenance of suboptimal blood pressure reflects a healthy cardiovascular system, which is a cooperative result of many biochemical and physiological factors, with the major control mechanism of blood pressure homeostasis being the classical renin-angiotensin–aldosterone system (RAAS) in the kidney. Endothelial nitric oxide (NO) plays an independent role in reducing the systemic blood pressure via increasing blood flow and oxygen delivery, through its vasodilator effect [1,2,3]. Furthermore, it exhibits anti-atherogenic properties, such as prevention of platelet adhesion and the aggregation and inhibition of proliferation and migration of leukocytes, endothelial cells, and vascular smooth muscle cells [1]. In times of deficient NO bioavailability, the properties and the functions of the vascular endothelium are modified, resulting an impaired vasodilation, creating a proinflammatory and prothrombic status, a condition identified as endothelial dysfunction, which later extends to cardiovascular diseases (CVD) [2]. It has further been reported that renal endothelium-derived NO might play a key role in pressure-dependent renin (RAAS initiating hormone) release from the liver [3,4].

NO is a metabolite of L-arginine, synthesized by the activity of endothelial NO synthase (eNOS), and its biosynthesis is mainly impaired due to oxidative stress-mediated endothelial L-arginine/NO pathway dysfunction [1,5,6]. Under oxidative stress conditions, the reaction of NO with superoxide ion (O^2•−^) forming peroxynitrite (ONOO^−^), a toxic and strong oxidant known to initiate organ dysfunction, diminishes its bioavailability [7]. Additionally, downstream metabolites of peroxynitrite, such as nitrotyrosine, impair endothelial function, modify the response of systemic arteries to angiotensin II and affect angiogenesis when bound to tubulin [7].

Homocysteine (Hcy), an intermediate metabolite of hepatic trans-methylation/trans-sulfuration pathways can impair the eNOS function and NO bioavailability, thus directly contributing to vascular dysfunction [8]. Hcy enhances reactive oxygen species (ROS)-mediated scavenging of NO by triggering the biosynthesis of superoxide ions via protein kinase C activation, in addition to its ability to block L-arginine transport to the endothelium and diminish the function of glutathione peroxidase; thus, it is directly associated with low bioavailability of NO [9,10]. Apart from its negative effect on NO bioavailability, due to its functions in ROS synthesis and paralyzing the intrinsic antioxidant defense mechanism of glutathione peroxidase, its higher serum concentration is a biomarker of increased oxidative stress and is thus considered as an independent risk factor for CVD [8]. Thus, maintenance of non-toxic levels of Hcy is important in the management of cardiovascular health [8].

Betaine (syn: trimethyl glycine), a quaternary ammonium compound, is known to play a key role in re-methylation of Hcy due to its ability to donate a single methyl group. Thus, it contributes to the maintenance of non-toxic levels of systemic Hcy and enhances levels of *S*-adenosylmethione (SAM), a strong antioxidant [11,12,13]. Further, previous studies have reported 54 to 80% reductions in Hcy levels upon 0.5% (low-dose) and 1.5% (high-dose) betaine supplementation [14]. During the past few decades, many studies have reported improved clinical conditions of arteriosclerosis, rheumatic disease, congestive heart failure, and hypertension upon betaine intervention, attributed to betaine’s methyl donor trait [9]. In this study, we hypothesized that dietary intake of betaine might improve NO bioavailability and subsequently ameliorate hypertension via its vasodilatory functions. Thus, the aim of the present study was to observe the beneficial effects of exogenous betaine supplementation (1%) on hypertension-prone groups, such as spontaneously hypersensitive (SHR) rats. Previous studies that used Wistar Kyoto (WKY) rats reported successful results in other research scenarios, such as non-alcoholic fatty liver disease, using 1% betaine dosage [15,16].

## 2. Materials and Methods

### 2.1. Materials

Betaine (anhydrate; minimum purity 99%; Nitten Betaine^TM^) extracted from sugar beet (*Beta vulgaris* subsp. vulgaris L.) was provided by Nippon Beet Sugar Mfg. Co., Ltd. (Obihiro, Japan). 

### 2.2. Animal Experimental Design and the Experimental Diets

Seven-week-old male WKY rats (*n* = 12) and SHR rats (n = 10) were purchased from Charles River Laboratories Japan Inc. (Yokohama, Japan). Rats were acclimatized on a commercial diet (CE-2, CLEA Japan, Inc., Tokyo, Japan) for 1 week and were grouped into four similar bodyweight (WKY, ≃160 g; SHR, ≃200 g) groups. Following the grouping, rats were fed with experimental diets (Table 1) with *ad libitum* access to water. Two groups (one group from each rat breed) were fed a control (CON) diet (WKY-con and SHR-con; n = 6 and n = 5, respectively) and the other two groups received a 1% betaine diet (WKY-b and SHR-b; n = 6 and n = 5, respectively). All diets were prepared according to AIN-76 diet guidelines. Each rat was housed individually and a feeder (≃25 g) and a drinker (≃150 mL) were allocated to each animal, which were replenished every day. The cages were maintained at 23 ± 1 °C temperature and 60 ± 5% relative humidity under a 12 h light/dark cycle. Systolic blood pressure (SBP), diastolic blood pressure (DBP), and mean blood pressure (MBP) were measured weekly between 09:00 to 12:00 h using the tail-cuff method (BP-98A-L, Softron, Tokyo, Japan) on conscious animals (pre-heated to 37 °C) according to the manufacturer’s instructions. After the experimental period of 8 weeks, the animals were sacrificed (sodium pentobarbital, 40 mg/kg body weight, Abbott Laboratories, Chicago, IL, USA) assuring a minimum level of suffering, and the blood was drawn from the intraperitoneal vein and kidney. The animal experiment was conducted according to the guidelines in the Guide for the Care and Use of Laboratory Animals. All procedures performed in the studies involving animals were in accordance with the ethical standards of the institution (Animal Care and Experiment Committee of Obihiro University of Agriculture and Veterinary Medicine, License no: 27-86).

### 2.3. Biochemical Analysis

Total cholesterol (TC), HDL cholesterol (HDL-C), triglyceride (TG), phospholipid levels, alanine aminotransferase (ALT), and aspartate aminotransferase (AST) in the serum were analyzed (Hitachi 7070 auto analyzer system, Tochigi, Japan). Angiotensin I converting enzyme (ACE) expression (Cloud-Clone Co., Houston, TX, USA) and serum NO levels (NO_2_/NO_3_ assay; Dojindo Laboratories, Mashiki-machi, Kumamoto, Japan) were measured using commercial assay kits. Serum and kidney glutathione (GSH) levels were determined as previously described [17]. Oxidative stress marker levels in kidney were analyzed using thiobarbituric acid reactive substances (TBARS) assay [18].

### 2.4. Statistical Analysis

Data were analyzed for their significance between control (WKY-con and SHR-con; n = 6, n = 5, respectively) and betaine-supplemented (WKY-b and SHR-b; n = 6, n = 5, respectively) groups of each rat breed by independent *t*-test in SPSS statistical software (IBM Co., Armonk, NY, USA) and are presented as mean ± standard error (SE). Pre-requisite statistical tests were conducted as specified with the SPSS software prior to actual statistical analysis using independent *t*-test. A two-way ANOVA using SPSS was conducted to identify potential interaction effects of rat species and betaine supplementation on blood pressure and biochemical data. Further, Pearson’s correlation analysis was conducted between serum biochemical parameters and blood pressure parameters using SPSS.

## 3. Results

### 3.1. Blood Pressure Parameters

**Figure 1:**
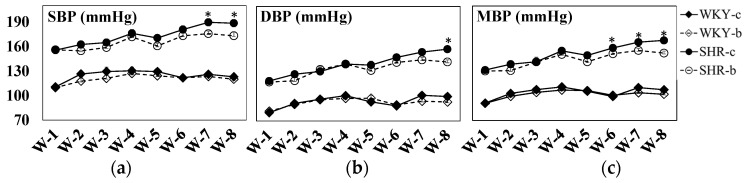
Changes in blood pressure parameters in SHR and WKY control rats (SHR-con, WKY-con) and betaine-supplemented rats (SHR-b, WKY-b): (**a**) systolic blood pressure (SBP); (**b**) diastolic blood pressure (DBP); (**c**) mean blood pressure (MBP). Data presented are average ± SE; n = 5, SHR and n = 6, WKY. Significant differences between the control (SHR-/WKY-con) and betaine-supplemented (SHR-/WKY-b) groups are represented by the ‘*’ at *p* < 0.05 as determined by the independent *t*-test.

Systolic blood pressure in the SHR-b group was significantly lower at weeks 7 (*p* = 0.020) and 8 (*p* = 0.013), while DBP at week 8 (*p* = 0.011) and MBP at weeks 6 (*p* = 0.046), 7 (*p* = 0.040), and 8 (*p* = 0.010) were also significantly lower compared with the respective control group (Figure 1). None of the parameters were significantly different between the two WKY groups. Furthermore, at weeks 7 and 8, a significant (*p* < 0.05) effect of betaine supplementation on the improvement of the three blood pressure parameters was revealed by two-way ANOVA analysis (Table 2). 

### 3.2. Biochemical Profile in Serum and Kidney

Serum NO levels in both SHR and WKY rats were significantly (*p* < 0.05) higher in the respective betaine-supplemented groups compared with their control groups (Table 3). On the other hand, serum ACE concentration was significantly (*p* < 0.05) lower in the SHR-b group compared with SHR-con, while the ACE concentration was only comparatively lower in the WKY-b group compared with the control group (Table 3). Serum GSH level was significantly (*p* < 0.05) lower in the SHR-b group compared with the SHR-con group, while it was similar between the WKY groups (Table 3). 

In both the WKY and SHR groups, TC and HDL-C were significantly (*p* < 0.05) higher in the betaine-supplemented groups, while non-HDL-C was significantly higher only in the SHR-b group (Table 3). A significantly (*p* < 0.05) lower serum TG level was observed in the WKY group upon betaine supplementation, whereas it was not significantly different between the SHR groups. In contrast, phospholipid level was significantly (*p* < 0.05) increased upon betaine supplementation in the SHR group. 

Serum AST and ALT levels were significantly (*p* < 0.05) higher in the WKY-con group compared to the WKY-b group but maintained within the healthy basal levels (Table 3). Levels of these enzymes were not significantly different between the SHR groups. The level of oxidative stress markers in kidney measured via TBARS was significantly (*p* < 0.05) lower in the SHR-b group compared with the SHR-con group, while it was not significantly different between the WKY groups (Table 3). 

The two-way ANOVA data presented in Table 4 indicate that the 1% betaine intervention and the inherent traits of the rat breeds used had significant (*p* < 0.05) main effects on determining the biochemical parameters analyzed in Table 3. However, there were no significant interaction effects except for ACE, which showed weak evidence for a potential interaction effect. Furthermore, as per the respective *p* values, the strength of the main effect of 1% betaine intervention on NO and ACE levels in serum seemed to be higher than that of the effect of the inherent traits of the rat breeds used. On the other hand, for serum lipid parameters, where both the main effects were significant, the inherent traits of the rat breeds seem to have had a higher effect than the betaine intervention.

Pearson’s correlation analysis revealed the presence of a significant (*p* < 0.05) negative correlation effect between serum NO level and the blood pressure parameters (week 8) in the SHR group (Table 5). Moreover, there was clearly a positive correlation between serum ACE level and the blood pressure parameters at week 8, despite being statistically non-significant.

## 4. Discussion

The key outcome of this study is that the SHR rats given a dietary supplement of 1% betaine exhibited significantly (*p* < 0.05) lower blood pressure by weeks 7 to 8. SHR rats are commonly used as an inbred genetic research model, having characteristics comparable with essential hypertension in humans. As the name suggests, these animals develop hypertension spontaneously at the age of 6 to 7 weeks, which reaches a stable level of hypertension by 17 to 19 weeks of age [19,20]. Several factors have been identified to ameliorate the high blood pressure in SHR rats, such as treatment with inhibitors of the RAAS, removal of the renal nerves, and treatment with antioxidants [20]. Thus, it has been postulated that the root causes of the development of hypertension in SHR rats may be associated with the over-activation of the RAAS through stimulated renin release from the kidneys and enhanced oxidative stress due to the activity of NADPH oxidase [20]. Thus, it is plausible to state that betaine might have been involved in one or both of the above-mentioned mechanisms to significantly lower blood pressure in the betaine-supplemented SHR rats, which was also statistically (Table 2) proved by the significant (*p* < 0.05) effect of betaine on SBP, DBP, and MBP at weeks 7 and 8.

Significantly lower ACE expression (58% reduction) in SHR-b rats could also have played a role in the blood pressure amelioration, albeit the Pearson’s correlations were not significant at the *p* < 0.05 level. ACE is the major enzyme that can regulate the biological actions of the RAAS through its action of cleaving two amino acids from biologically inactive angiotensin I to form its biologically active form, which in fact the major active peptide in the RAAS, angiotensin II [21,22]. Angiotensin II is responsible for the regulation of salt balance and the blood pressure by retaining water/salt and increasing blood pressure through its vasoconstriction functions [21,23]. Thus, abnormal activation of the RAAS leads to the development of hypertension, cardiac hypertrophy, and finally, heart failure in general [24]. Blocking angiotensin II synthesis using ACE inhibitors is considered as the main pharmacological intervention in the management of hypertension and related pathologies. However, in this study, the significantly lower expression of ACE in the betaine-supplemented SHR rat group (Table 3) could have been due to an inhibitory effect of betaine on ACE expression, as similarly suggested by the two-way ANOVA analysis (Table 4).

The significant effect of betaine supplementation on lowering serum ACE expression as per the two-way ANOVA analysis (Table 3 and Table 4) was in agreement with previous findings [21,22,25]. A bidirectional and counter-regulatory association has been reported between eNOS expression and ACE expression [22]. A study on cardiomyopathic Syrian hamsters, an animal model with a genetic predisposition to endothelial dysfunction attributed to its reduced expression of eNOS, reported an increase in eNOS expression upon chronic treatment with an ACE inhibitor [26]. Additionally, attenuation of the expression and activity of ACE via sodium nitroprusside (NO donor) and L-arginine (substrate for NO synthesis) and ACE expression enhancement via suppressed NOS expression further support the counter-regulatory association between ACE expression and NO bioavailability [27,28]. In this study, negative Pearson’s correlation coefficients were observed between serum NO and ACE levels in both SHR-b and WKY-b groups, despite being non-significant.

The observed lower blood pressure could be directly attributed to the vasodilatory functions and other vasoprotective functions of NO that act independently of systemic and local RAAS [1,2,29]. NO is considered as a nitrovasodilator drug homologue, which can stimulate the cellular levels of cyclic guanosine monophosphate in smooth muscles of the heart, leading to vasodilation [28,29]. The above fact is further supported by the significantly higher NO level observed in the betaine-supplemented SHR rat group. Moreover, it is further strengthened by the significant correlation between NO and blood pressure parameters (Table 5). Despite the significantly higher serum NO level observed in the WKY-b group, the absence of a significant correlation between NO and blood pressure parameters further clarifies the similar blood pressure parameters observed between WKY and WKY-b. Enhanced bioavailability of NO can be attributed to betaine’s function in re-methylation of Hcy, thus mitigating Hcy’s inhibitory effects on NO bioavailability [6,30,31]. Hyperhomocysteinemia is known to restrict NO bioavailability by influencing ROS bioavailability (through the activity of NADPH oxidase), inhibiting eNOS function (through activating protein kinase C and inhibiting the activity of the dimethylarginine dimethylaminohydrolase enzyme), inhibiting the transport of L-arginine required for NO synthesis by methylation and inhibiting the activity of glutathione peroxidase [6,11,30,32,33].

Betaine’s function in detoxification of Hcy via re-methylation is also reflected in the significantly lower kidney TBARS value in the SHR-b rat group, although the serum Hcy level was not measured in this study, which is a limitation [10,34]. As an estimate of the lipid peroxidation state, a higher value of TBARS is a biomarker of oxidative stress, which is known to cause organ dysfunction and essential hypertension [35]. Oxidative stress eventuates due to the accumulation of ROS (free radicals and/or their metabolites) as a result of increased ROS production and/or diminished intrinsic antioxidant mechanisms [35]. Hcy is a well-known independent risk factor for vascular oxidative stress and endothelial dysfunction through enhanced ROS bioavailability and ROS production attributed to inhibited glutathione peroxidase activity and protein kinase C activation, respectively. Enhanced bioavailability of ROS ultimately leads to reduced bioavailability of NO, attributed to the scavenging effect on NO [8]. Thus, comparatively lower serum liver enzyme levels also support the low oxidative stress status, as similarly implicated by the significantly lower TBARS in the SHR-b rat group [36]. The higher levels of serum liver enzymes observed in the two SHR rat groups compared with the basal levels (AST: 50–150 IU/L; ALT: 30–130 IU/L) could have been due to their genetic predisposition to hypertension, as a relationship between hypertension and elevated levels of serum liver enzymes has been reported [37,38].

GSH is a major endogenous, non-enzymatic antioxidant defense against oxidative stress and previous reports have reported a close direct relationship between lower systemic levels of GSH and progression of oxidative stress-induced cardiac diseases [10,39,40,41]. However, the 42% lower serum GSH level observed in the betaine-supplemented SHR rat group could be attributed to the betaine’s function in re-methylation of Hcy in the kidney and liver, instead of trans-sulfuration to synthesize cysteine, the substrate for GSH synthesis. Significantly lower kidney GSH levels in the SHR-b rat group also support the above fact, as the kidneys serve as the major site for plasma GSH clearance [42]. Furthermore, higher bioavailability of NO is known to downregulate the activity of cystathione β-synthase, the enzyme that catalyzes the initial step of the trans-sulfuration pathway to convert Hcy to cysteine, which can also lead to less synthesis of GSH and subsequently less serum GSH content [11].

Consequently, significantly higher NO bioavailability resulted from potentially lower ROS in the circulation as a result of the re-methylation action of betaine on Hcy, and the significantly lower expression of ACE that resulted as a counter-regulatory action of enhanced eNOS expression (manifested by the higher NO in the circulation) in the betaine-supplemented SHR rats might have acted independently and as well as integratively to ameliorate the high blood pressure. Thus, the observations from this study suggest a positive therapeutic effect of betaine on the management of hypertension in high-risk groups represented by the genetically predisposed SHR rats.

An increased serum lipid profile is considered as an undesirable effect, as it is indicative of hypercholesterolemic status, a classic risk factor for oxidative stress-mediated atherosclerotic cardiovascular events [6]. To date, the findings on the relationship between dietary betaine intake and serum lipid profile are inconsistent, where both betaine deficiency and higher betaine intake have been reported to be associated with higher prevalence of hypercholesterolemia [43]. Although the effect of betaine on lipid metabolism is not well established, it has been suggested that betaine may cause an increment in blood lipid levels as a result of the increased export of lipids in the circulation attributed to the lipotropic effects of betaine through enhanced very-low-density lipoprotein (VLDL) secretion, as implicated by significantly higher non-HDL-C (VLDL, intermediate-density lipoproteins, low-density lipoproteins, chylomicrons, and lipoprotein remnants) in both betaine-supplemented groups [7,34]. Both SAM (an intermediate of the trans-methylation pathway) concentration and betaine homocysteine methyltransferase (an enzyme catalyzing Hcy re-methylation by betaine) expression are considered to be enhanced by betaine supplementation, which in turn is considered to influence the synthesis of phosphatidylcholine (PC), a vital component of VLDL, via methylation of phosphatidylethanolamine (PE), thus enhancing secretion of VLDL into the circulation, reducing the risk of fat accumulation in the liver [7]. Elimination of methionine and choline, the two methyl donors for PC synthesis, from a hepatocyte culture medium led to reduced VLDL secretion [44]. Previous studies have reported that phosphatidylethanolamine N-methyltransferase (catalyzes methylation of PE; PE + methyl → PC) knockout mice exhibited 50% reduction in TG secretion (as VLDL) from hepatocytes causing accumulation in the liver compared with their wild-type counterparts [15,44,45]. Thus, enhanced bioavailability of methionine and SAM attributed to the betaine’s re-methylation effect on Hcy might have improved VLDL synthesis and secretion from the liver, thus preventing accumulation [46,47]. Excessive TG accumulation in the liver leads to the development of hepatic steatosis, the initial stage of the chronic kidney disease spectrum [48]. A previous study that supplemented dietary betaine (1%) to a non-alcoholic steatohepatitis mouse model revealed an ameliorative effect on hepatic steatosis [49]. It has been reported that betaine improves non-alcoholic fatty liver disease by decreasing the serum Hcy level and enhancing the hepatic SAM level [50].

In conclusion, oral supplementation with 1% betaine significantly lowered blood pressure in the SHR group, potentially due to the improved serum NO bioavailability, attributed to the vasodilatory functions of NO. Furthermore, prevention of over-activation of RAAS, as implicated by the lower expression level of ACE, could also have contributed to the observed amelioration of hypertension. Circulatory ACE expression might have been reduced by the higher eNOS activity, as manifested by the higher circulatory NO, in a counter-regulatory manner. Although betaine supplementation caused higher serum cholesterol levels, it appears that its negative effects might have been mitigated by the Hcy detoxifying function of betaine (low ROS as reflected by low TBARS), the vasoprotective effects of improved bioavailability of NO (due to detoxification of Hcy by betaine), and lower ACE expression, as manifested by the significantly lower blood pressure. Moreover, the putative serum non-HDL-C enhancing effect may also reflect a hepatoprotective effect through the prevention of fat accumulation in liver via betaine’s lipotropic effect, which involves enhanced VLDL secretion. However, further studies should be conducted to determine the long-term net outcomes of the serum-lipid-enhancement and Hcy-lowering effects of betaine on cardiovascular and hepatic health.

## Figures and Tables

**Table 1:** Composition of control and betaine-supplemented diets fed to WKY and SHR rats.

Component	g/Kg	
	Control Diet	Betaine Diet
Milk casein	210	200
DL-methionine	3	3
Granulated sugar	500	500
Cellulose	50	50
Corn oil	50	50
Vitamin mix (AIN-76)	10	10
Mineral mix (AIN-76)	35	35
Choline bitartrate	2	2
Betaine	-	10
Corn starch	140	140

**Table 2:** Two-way ANOVA analysis for SBP, DBP, and MBP.

BP Parameter	SBP		DBP		MBP	
Week	W-7	W-8	W-7	W-8	W-7	W-8
Betaine	0.015	0.005	0.030	0.005	0.016	0.003
Species	<0.001	<0.001	<0.001	<0.001	<0.001	<0.001
Interaction	0.082	0.045	0.746	0.218	0.525	0.125

**Table 3:** Serum and kidney biochemical parameters of WKY and SH rats.

Parameter	WKY			SHR		
	WKY-con	WKY-b	*p* Value	SHR-con	SHR-b	*p* Value
	Serum					
NO (µmol/L)	20.57 ± 2.50	37.59 ± 6.35	0.032	13.20 ± 2.51	26.76 ± 4.76	0.036
ACE (ng/mL)	3.15 ± 0.43	2.35 ± 0.52	0.265	5.99 ± 1.06	2.50 ± 0.57	0.020
GSH (nmol/g)	56.10 ± 10.51	58.44 ± 12.44	0.899	77.19 ± 8.27	44.55 ± 7.89	0.021
TC (mmol/L)	3.96 ± 0.15	4.41 ± 0.12	0.041	1.88 ± 0.06	2.45 ± 0.17	0.015
HDL-C (mmol/L)	0.82 ± 0.03	0.91 ± 0.03	0.048	0.45 ± 0.01	0.54 ± 0.03	0.020
Non-HDL-C (mmol/L)	3.14 ± 0.12	3.50 ± 0.12	0.063	1.43 ± 0.05	1.91 ± 0.15	0.015
TG (mmol/L)	2.18 ± 0.06	1.76 ± 0.14	0.022	2.20 ± 0.21	2.63 ± 0.54	0.482
PL (mmol/L)	6.85 ± 0.25	7.62 ± 0.15	0.025	4.18 ± 0.14	5.09 ± 0.32	0.030
AST (IU/L)	99.50 ± 3.64	79.50 ± 2.83	0.001	204.60 ± 21.45	188.60 ± 38.59	0.726
ALT (IU/L)	26.33 ± 0.84	21.50 ± 1.34	0.012	86.40 ± 29.02	110.60 ± 40.62	0.641
	Kidney					
GSH (nmol/g)	248.85 ± 6.58	247.53 ± 2.51	0.856	217.37 ± 7.04	221.28 ± 5.56	0.675
TBARSs (nmol/g)	140.39 ± 3.77	129.58 ± 8.02	0.251	68.60 ± 1.98	60.52 ± 1.71	0.015

Abbreviations: NO, nitric oxide; ACE, angiotensin I converting enzyme; GSH, glutathione; TC, total cholesterol; HDL-C, HDL cholesterol; TG, triglycerides; PL, phospholipids; AST, aspartate aminotransferase; ALT, alanine aminotransferase; TBARS, thiobarbituric acid reactive substances.

**Table 4:** Two-way ANOVA analysis of biochemical parameters at week 8.

Component	Two-Way ANOVA *p* Value		
	Species	Betaine	Species × Betaine
Nitric oxide	0.058	0.003	0.705
Angiotensin I converting enzyme	0.036	0.004	0.056
Serum glutathione	0.732	0.162	0.109
Total cholesterol	<0.001	0.001	0.651
HDL cholesterol	<0.001	0.003	0.995
Non-HDL cholesterol	<0.001	0.002	0.617
Triglycerides	0.121	0.984	0.144
Phospholipids	<0.001	0.002	0.777
Aspartate aminotransferase	<0.001	0.382	0.922
Alanine aminotransferase	0.004	0.673	0.528
Kidney TBARS	<0.001	0.077	0.791
Kidney glutathione	<0.001	0.820	0.648

Significant (*p* < 0.05) interaction and main effects of species and betaine supplementation as determined by the two-way ANOVA.

**Table 5:** Pearson’s correlations between blood pressure parameters at week 8 and serum NO and ACE levels.

Parameter 1	Parameter 2	SHR	WKY
NO	SBP	−0.590 (*p* = 0.043)	0.095 (*p* = 0.770)
	DBP	−0.585 (*p* = 0.046)	−0.191 (*p* = 0.552)
	MBP	−0.595 (*p* = 0.041)	−0.147 (*p* = 0.649)
ACE	SBP	0.494 (*p* = 0.103)	−0.299 (*p* = 0.345)
	DBP	0.556 (*p* = 0.060)	−0.077 (*p* = 0.811)
	MBP	0.543 (*p* = 0.068)	−0.103 (*p* = 0.750)

Significant (*p* < 0.05) correlation effects as determined by the Pearson’s correlation analysis.

## Data Availability

The raw data supporting the conclusions of this article will be made available by the authors on request.
